# Association of Parental Famine Exposure With Offspring Depression and Cognition Function

**DOI:** 10.3389/fpsyt.2022.812805

**Published:** 2022-04-05

**Authors:** Ye Liu, Yu Liu, Yuzhu Lu, Jiangping Li, Shulan He

**Affiliations:** ^1^Department of Epidemiology and Health Statistics, School of Public Health and Management, Ningxia Medical University, Yinchuan, China; ^2^Key Laboratory of Environmental Factors and Chronic Disease Control, Yinchuan, China

**Keywords:** famine, depression, cognition, inheritance, early-life exposure

## Abstract

**Background:**

The effect of early exposure to famine on depression and cognition in adulthood has been shown, but the intergenerational association of famine remain to be explored. This study focused on exploring the association of parental famine exposure with depression and cognition in the offspring.

**Methods:**

Based on the Chinese Family Panel Studies database, which is a longitudinal survey, we included 5,150 individuals born between 1959 and 1961 and divided them into fetal-exposed, infancy-exposed (birth year = 1957–1958), school-age-exposed (birth year = 1949–1956), adolescent-exposed (birth year = 1946–1948), and unexposed groups. We used one-way analysis of variance, multiple linear regression, and one follow-up measurement to analyze the association between parental famine exposure and offspring depression and cognitive function.

**Results:**

Compared with the unexposed group, the correlations between parental famine exposure during fetal period and their cognitive function (mother: β = –1.614, 95% CI: –2.535, –0.693; *p* = 0.001; father: β = –2.153, 95% CI: –3.104, –1.202, *p* < 0.001) were significant. For the offspring, there was a negative correlation between famine exposure of fathers during the fetal period and depression in their offspring (β = –0.477, 95% CI: –0.907, –0.047; *p* = 0.030). There was a negative correlation between maternal famine exposure during the infant and adolescent period and cognitive function in the offspring (math test: β = –0.730, 95% CI: –1.307, –0.153; *p* = 0.013; word test: β = –2.346, 95% CI: –4.067, –0.625; *p* = 0.008).

**Limitations:**

Not all variables related to depression and cognition function were included in the CFPS database, and the other unknown or unmeasured confounders may explain our results.

## Introduction

Depression has gradually attracted the attention of the world because of its great physical and mental damage to patients. According to research statistics, the global 12-month prevalence rate of major depressive disorder is approximately 6%, with a lifetime risk of approximately 18% (1). It is expected that depression will rank first among the global disease burden by 2030. Depression has complex causes and many risk factors, including cognitive impairment, stress, and intergenerational factors (2).

Mild cognitive impairment (MCI) describes people with symptoms that are between normal age-related cognitive decline and dementia. The characterization of MCI was defined by Petersen et al. (3) as the presence of subjective memory complaints, intact ability to carry out everyday activities such as meal preparation, preserved general cognitive abilities, objective evidence of a memory deficit, and the absence of dementia. The negative effects of cognitive impairment on individuals should not be underestimated. In China, the prevalence of mild cognitive impairment in people over the age of 60 is approximately 15.5% (4), and the well-known risk factors are age, history of parental dementia, and poor education. Cognitive impairment is one of the core features of depression; about two-thirds of patients with depression also exhibit cognitive impairment (5). The most frequently reported cognitive impairments associated with depression were deficits in: attention, executive functions, memory, and processing speed (6). Significantly, lower hippocampal volumes in the depressed group than among non-depressed controls have been reported (7). A meta-analysis showed that patients with depression have a greater degree of cognitive impairment in executive and memory functions than healthy people (8).

Malnutrition is one of the major risk factors affecting brain development and mental health, increasing the risk of developing depression and cognitive impairment. There is evidence that malnutrition during pregnancy is associated with poorer cognitive function in the offspring (9). Studies in rodents have shown that increasing risk of cognitive disorder is associated with perinatal malnutrition through structural, functional, and molecular changes in brain development (10). Additionally, malnutrition in an individual during the fetal period and even in the early stages of life is also a risk factor for depression. According to the China Health and Retirement Longitudinal Study, the Chinese Great Famine accounted for 13.6% of the depressive symptom burden (11).

From 1959 to 1961, an extreme and widespread food shortage occurred in China. During this period, the number of abnormal deaths in China was conservatively estimated to be approximately 17 million, with the majority among the rural population, in 1960, when the famine was at its worst; for example, the abnormal mortality rate was as high as 28 per thousand, about twice as high as in cities (12). This large-scale famine directly led to individual malnutrition. A large number of studies have focused on the effects of famine exposure in early life on health in adulthood, such as cerebral hemorrhage (13), dyslipidemia (14), abortion (15), premature menopause (16), and increased risk of diseases such as metabolic syndrome (17, 18). It is worth noting that most studies have been limited to the effects of famine on a single generation, while few studies have explored the intergenerational impact of famine exposure on the health of the offspring of those exposed to famine.

An important strand of this study explores the intergenerational association between parental famine exposure and offspring mental health. Adverse prenatal and postnatal living conditions may affect health trajectories across the lifespan and into future generations based on the key concept of “Developmental Origins of Health and Disease (DOHaD).” Some researchers believe that famine might lead to an intergenerational risk of diabetes by epigenetic modifications resulting from poor nutrition during the critical window of early development in utero (19). The effects of famine exposure on cognitive function and depression have been reported for one generation. However, there are only a few studies on the intergenerational association of famine with depression and cognition, which is a major innovation in our study.

In this study, the Chinese Family Panel Studies (CFPS) 2010 and 2018 databases were used to explore the association between parental famine exposure and depression and cognition in offspring. We hypothesized that parental exposure to the famine of 1959–1961 in China will increase the risk of depression and cognitive impairment in offspring.

## Method

### Data Source and Sampling

Families and individuals from the 2010 and 2018 CFPS databases were used as subjects. CFPS is a longitudinal survey launched by Peking University in 2010. The CFPS aims to reflect the changes in society, economy, population, education, and health in China by tracking and collecting three levels of data: individuals, families, and communities, and provides a data basis for academic research and public policy analysis (20). An implicit stratified, multi-stage Probability-Proportional-to-Size Sampling (PPS) were used to obtain the samples from the 2010 CFPS baseline survey, which covered 25 provinces (nearly 16,000 households) in China, representing 94.5% of China’s population (21), and has the characteristics of being large scale and possessing strong comprehensiveness.

To assess the association between parental famine exposure and offspring depression and cognitive function, we included all the data from households in the 2010 CFPS. Among them, families that did not contain a parental-offspring relationship were excluded. We excluded 27,151 individuals: (1) an individual with a family of one member; (2) an individual with two family members, and the relationship is husband and wife, brother and sister, or grandparents and grandchildren; and (3) an individual with three or more family members who are siblings or grandparents and grandchildren. Then, we matched the rest of the 2010 CFPS data with the 2018 CFPS data. As a result, a total of 5,150 participants were included in the analysis.

**FIGURE 1:**
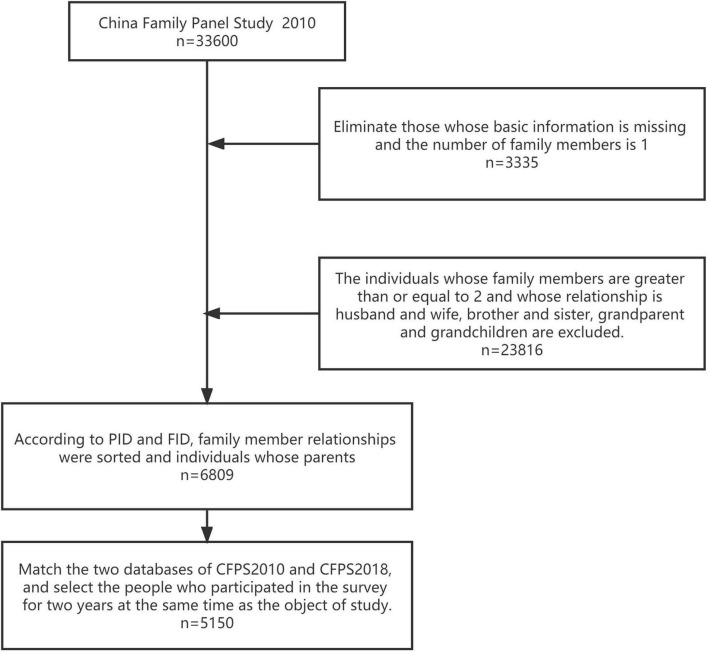
Flow chart of patient inclusion. A total of 33,600 respondents participated in the survey in the 2010 CFPS database used in the study. Families that did not contain a parental-offspring relationship were excluded. After matching the rest of the 2010 CFPS data with the 2018 CFPS data, a total of 5,150 participants were included.

The screening process is shown in Figure 1.

### Categories of Famine Exposure

The inclusion criteria of the exposure group were parents and their offspring who participated in both the 2010 and 2018 surveys, and parents who were exposed to famine in 1959–1961, from in utero to puberty. The famine group was divided into five groups according to the birth time of the parent, which were as follows:

1) born 1946.1.1–1948.12.31 are classified as adolescent famine exposure;
2) born 1949.1.1–1956.12.31 are classified as school-age famine exposure;
3) born 1957.1.1–1958.12.31 are classified as infancy famine exposure;
4) born 1959.1.1–1961.12.31 are classified as fetal famine exposure;
5) born 1962.1.1–1969.12.31 are classified as unexposed group.

### Depressive Symptoms

Depression was measured using two scales: the 2010 questionnaire used The Kessler Psychological Distress Scale (K6) (22), the 2018 questionnaire used the American Center for Epidemiological Research Depression Scale (CES- D8) (23). CFPS, 2010 contains six items related to feelings in past month. The respondents were asked to indicate how often they had those feelings from the five options–“almost every day,” “often,” “half the time,” “sometimes,” and “never.” The five options correspond to 1, 2, 3, 4, 5 and the possible total score ranges from 6 to 30. The score used for factor analysis is the score of individual depression; the lower the score, the more serious the degree of depression. According to the official manual, the cutoff value of factor analysis score is 0, the score <0 is depression and >0 is normal (24). The Cronbach’s alpha coefficient of this table was 0.855 (25), indicating high reliability. CES- D8 contains eight items to describe a list of fillings, which were six positive and two negative questions. The respondents were asked to indicate their felling with a 4-point scale using this scale. The total score was from 8 to 32 and a higher score indicated a higher level of depressive symptoms, the total score >15 is depression, and ≤15 is normal (26). According to the suggestion of the CFPS, we used the percentile method to perform the equivalent operation on the scores of the two sets of questions.

### Cognitive Symptoms

In the 2010 and 2018 CFPS, the self-written literacy test and the comprehensive score of the math test were used to measure the cognitive level of the interviewees (23). The theoretical basis of the cognitive test was the Guttman test in psychometry, which has good reliability and validity (18). These two sets of questions examine the word memorization (word test) and mathematical calculation abilities (math test), the higher the score, the better the cognitive level. In the 2018 CFPS, cognitive scores were generated for respondents assuming a fixed starting point to ensure comparability with the 2010 CFPS (23).

The literacy test consisted of 34 Chinese characters and was sorted in ascending order of difficulty. Moreover, the test had different starting points according to the academic degree of the interviewee to increase efficiency. The CFPS research group assumed that respondents with junior high school education knew at least eight words, and respondents with high school education or above knew at least twenty words. If the degree is elementary school or below, the questions will be asked in order from the first character; if the degree is junior high school, the starting point will be the ninth character, with the questions asked in order; if the interviewee has a high school education or above, they will start from the 21st character, with the questions asked in order. If the respondent fails to answer a question correctly, the question number of the question before the starting question is used as the score (25).

The scoring method of the math test was similar to that of the literacy test. The starting point is still determined by the subject’s academic level. If the academic qualification is elementary school or below, the questions will be asked in order from the first question; if the academic qualification is junior high school, the questions will start with question 5, with the questions asked in order; if the interviewee has a high school education or above, they will start with question 13, with the questions asked in order. If the respondent fails to answer a question correctly, the question number of the question before the starting question is used as the score (25).

### Assessment of Covariates

The CFPS team collected data through face-to-face and telephone interviews, from which we selected variables, including current location, sex, education level, marital status, age in 2010, income level, social relation, happiness, social status, life satisfaction, body mass index (BMI), health status, diagnosed disease, smoking status, drinking status, and nap status.

Education level was divided into illiteracy/elementary and junior high school and above. Marital levels were grouped into unmarried/married, cohabiting/divorced, or widowed. In addition, income level, social relation, happiness, social status, and life satisfaction were determined by the subjective sensation of the respondents; the score ranged from 1 to 5, and the higher the score, the better the self-feeling. Health status is classified as healthy/general/relatively unhealthy/unhealthy/very unhealthy. Higher scores indicated worse conditions. For diagnostic diseases, responders were asked whether they had a chronic disease diagnosed by a doctor in the past 6 months. For smoking status, responders were asked whether they smoked or did not smoke in the past month. For drinking status, responders were asked whether they drank alcohol at least three times a week in the past month. For nap status, responders were asked whether they have nap habits.

### Ethics Statement

The Peking University Biomedical Ethics Review Committee provided ethical approval for the survey (Approval number: IRB00001052-14010).

### Statistical Analyses

Stata MP 15 software was used for data collection and statistical analyses. The basic characteristics of baseline participants were analyzed using the descriptive statistical data from 2010; the measurement data were expressed as mean ± standard deviation, and the counting data were expressed as number of cases and percentage.

One-way analysis of variance (ANOVA) was used to compare the groups with different degrees of parental famine exposure to analyze whether the parents’ different degrees of famine exposure was related to their own and their offspring in terms of depression scores, mathematical test scores, and word tests. Bonferroni multiple comparison analysis was used to understand subgroup differences among the different parental famine exposure groups. The data are expressed as the mean ± standard deviation, with an α set at 0.05.

Multivariable linear regression and logistic regression models were used to measure the correlation between famine-exposed parents and their offspring and their depression, and cognition scores, while adjusting for current location, sex, education level, marital status, age in 2010, income level, social relation, happiness, social status, life satisfaction, body mass index (BMI), health status, diagnosed disease, smoking status, drinking status, and nap status. The dependent variable was both parental and their offspring’s depression scores and cognition function scores, including the score of the math test and word test, respectively. Independent variables were the groups of parental famine exposure. The data of the 2018 offspring was corrected using the basic information and their parents’ depression scores in 2010. The α was set at 0.05.

To compare the differences between the baseline measurement in 2010 and the follow-up measurement in 2018 between the famine exposure group and the unexposed group, one follow-up measurement was used to analyze the scores of different parental exposure groups from 2010 to 2018, using the following formula:


$$△\,\text{Y}=\frac{\left({\text{Y}}_{\mu \,2}-{\text{Y}}_{\mu \,1}\right)}{\left({\text{Y}}_{\text{max}}-{\text{Y}}_{\text{min}}\right)}\times 100\%$$

In this method, the ceiling and floor effects are considered, and the variation in the difference with time is more accurately defined. Y_μ2_ refers to the observed value of individual i at time point t2, and Y_μ1_ refers to the observed value of individual i at time point t1. Y_max_ is the maximum possible value of Y (“ceiling effect”), and Y_min_ is the minimum possible value of Y (“floor effect”). In this study, Ymax and Ymin refer to the theoretical maximum and minimum scores of the math test and word test relative to scale, respectively. Thus, for math test, Ymax and Ymin were 24 and 0, respectively, and for word test Ymax and Ymin were 34 and 0, respectively.

## Results

Table 1 shows the basic characteristics of the participants in the baseline study. In 2010, 1,576 people had both parents who were exposed to famine, including 2,328 mothers and 2,235 fathers. In 2010, the average age of mothers was 49.8 years, the average age of fathers was 51.5 years, and the average age of the children was 24.5 years. Among the respondents, rural people accounted for a large proportion of the two generations, and there were more males among the offspring.

**TABLE 1:** 2010 general characteristics of study population according to family membership.

Variables	Mother	Father	Offspring
**Location, *n* (%)**			
Rural	2,551 (53.37)	2,298 (54.21)	2,776 (53.90)
Urban	2,229 (46.63)	1,941 (45.79)	2,374 (46.10)
**Sex, *n* (%)**			
Female	–	–	1,838 (35.69)
Male	–	–	3,312 (64.31)
**Education level, *n* (%)**			
Illiteracy	2,086 (43.69)	980 (23.13)	343 (6.66)
Elementary and junior high school	2,103 (44.04)	2,392 (56.46)	2,896 (56.25)
High school and above	586 (12.27)	865 (20.42)	1,909 (37.08)
**Marital level, *n* (%)**			
Unmarried	7 (0.15)	7 (0.17)	3,289 (63.86)
Married or cohabiting	4,424 (92.59)	4,101 (96.74)	1,769 (34.35)
Divorced or widowed	347 (7.26)	131 (3.09)	92 (1.79)
Age in 2010 (mean ± sd)	49.860 ± 6.944	51.537 ± 7.307	24.473 ± 6.491
Income level (mean ± sd)	2.102 ± 0.971	2.355 ± 0.952	2.237 ± 0.949
Social relation (mean ± sd)	4.025 ± 0.862	4.000 ± 0.849	3.964 ± 0.832
Happiness (mean ± sd)	3.811 ± 1.021	3.811 ± 1.001	3.898 ± 0.961
Social status (mean ± sd)	2.788 ± 0.974	2.807 ± 0.975	2.687 ± 0.893
Life satisfaction (mean ± sd)	3.456 ± 1.033	3.451 ± 1.037	3.448 ± 0.996
BMI (mean ± sd)	22.815 ± 3.268	22.853 ± 3.175	21.232 ± 3.191
Health (mean ± sd)	1.970 ± 1.039	1.789 ± 0.970	1.368 ± 0.651
Diagnosed disease, *n* (%)	898 (18.80)	674 (15.91)	239 (4.64)
Smoking, *n* (%)	193 (4.06)	2,768 (65.58)	1,579 (30.79)
Drinking, *n* (%)	157 (3.30)	1,412 (33.45)	657 (12.81)
Nap, *n* (%)	2,186 (45.93)	2,180 (51.65)	2,359 (46.00)

*sd, standard deviation.*

Table 2 shows that maternal late-life depression, math test and word test scores in maternal fetal-stage exposed group were significantly higher than that in adolescent-stage exposed group (*p* < 0.05), and the scores for depression and cognition were the lowest in mothers who were exposed to adolescent famine. Maternal late-life math test and word test score in maternal adolescent and school-age exposed groups were significantly lower than that in the non-exposed group (*p* < 0.05). A similar conclusion was obtained in the paternal famine exposure group, paternal late-life math test and word test scores in paternal adolescent and school-age exposed groups were significantly lower than that in the non-exposed group (*p* < 0.05); the cognitive level score was the lowest in fathers who were exposed to adolescent famine. Offspring math test and word test scores were significantly lower in maternal infant, school-age and adolescent-stage exposed groups than that in the non-exposed group (*p* < 0.05), while it was not significantly different in paternal infant-stage exposed group than that in the non-exposed group (*p* > 0.05).

**TABLE 2:** Univariate analyzes results about offspring in terms of the depression scores, math test scores, and word tests among their parents in different famine exposure level.

Group	Paternal			Offspring		
	Depression score	Math test	Word test	Depression score	Math test	Word test
**Mgroup**						
Adolescent	26.061 ± 4.751	4.519 ± 5.434	7.519 ± 9.706	27.633 ± 3.094	11.735 ± 6.257	20.244 ± 9.416
School-age	26.656 ± 3.986	5.585 ± 5.845	9.243 ± 10.172	27.337 ± 3.178	13.962 ± 5.682^a^	23.140 ± 8.236^a^
Infant	26.436 ± 4.041	7.943 ± 6.864^ab^	11.161 ± 10.516^ab^	27.233 ± 3.239	14.887 ± 5.715^a^	24.632 ± 7.580^ab^
Fetal	26.978 ± 3.734^a^	9.169 ± 6.727^ab^	13.540 ± 9.996^abc^	27.063 ± 3.142	16.155 ± 5.534^abc^	25.430 ± 7.020^ab^
Control	26.711 ± 3.720	8.803 ± 5.996^ab^	15.325 ± 9.902^abcd^	27.104 ± 3.060	16.245 ± 5.452^abc^	26.100 ± 6.848^abc^
F	2.71	81.99	96.69	2.68	64.88	58.37
*P*	0.0286	<0.001	<0.001	0.0300	<0.001	<0.001
**Fgroup**						
Adolescent	27.246 ± 4.199	7.202 ± 5.865	13.093 ± 10.825	27.630 ± 2.935	12.453 ± 6.122	21.240 ± 8.731
School-age	27.145 ± 3.916	9.214 ± 6.045^a^	15.866 ± 9.911^a^	27.217 ± 3.277	14.326 ± 5.735^a^	23.531 ± 8.067^a^
Infant	27.289 ± 3.404	11.892 ± 6.083^ab^	17.645 ± 9.302^ab^	27.412 ± 2.696	16.121 ± 5.291^ab^	25.787 ± 6.612^ab^
Fetal	27.397 ± 3.578	12.295 ± 5.961^ab^	18.327 ± 9.117^ab^	26.977 ± 3.185	15.773 ± 5.735^ab^	24.951 ± 7.817^ab^
Control	27.368 ± 3.380	11.412 ± 5.433^abd^	19.397 ± 8.514^abc^	27.191 ± 2.996	16.260 ± 5.391^ab^	26.234 ± 6.719^abd^
F	0.77	61.93	42.77	2.21	41.34	41.41
*P*	0.5442	<0.001	<0.001	0.0659	<0.001	<0.001

*^a^Significantly different from Adolescent group (p < 0.05).*

*^b^Significantly different from School-age group (p < 0.05).*

*^c^Significantly different from Infant group (p < 0.05).*

*^d^Significantly different from Fetal group (p < 0.05). Mgroup and Fgroup refer to the famine group according to mother’s or father’s birth time, respectively. Mothers or fathers who were born 1946.1.1–1948.12.31 are classified as adolescent famine exposure; born 1949.1.1–1956.12.31 are classified as school-age famine exposure; born 1957.1.1–1958.12.31 are classified as infancy famine exposure; born 1959.1.1–1961.12.31 are classified as fetal famine exposure; born 1962.1.1–1969.12.31 are classified as the control group.*

Table 3 shows that father’s fetal famine exposure was significantly associated with reduced scores for depression in their offspring in 2010 compared with the unexposed group (*p* = 0.030). Father’s fetal famine exposure increased the risk of developing offspring depression by 1.472 times (95%CI [1.044, 2.075]). Compared with the unexposed group, there were no statistically significant differences between parental famine exposure and depression in the offspring in 2018 (*p* > 0.05).

**TABLE 3:** Association of famine-exposed parents with their offspring and their depression scores from 2010 to 2018.

	Parental				Offspring^‡^			
	β (95%CI)	*P*	*aOR* (95%CI)	*P*	β (95%CI)	*P*	aOR* (95%CI)	*P*
**Survey year: 2010**								
**Mgroup**								
Adolescent	0.387 (–0.726, 1.500)	0.495	1.125 (0.551, 2.295)	0.746	0.501 (–0.356, 1.358)	0.252	0.588 (0.269, 1.285)	0.183
School-age	0.564 (–0.159, 1.286)	0.126	0.951 (0.598, 1.510)	0.830	–0.146 (–0.675, 0.382)	0.587	1.096 (0.710, 1.693)	0.679
Infant	0.170 (–0.427, 0.768)	0.577	1.073 (0.731, 1.575)	0.718	–0.117 (–0.647, 0.414)	0.666	1.066 (0.690, 1.646)	0.773
Fetal	0.482 (0.001, 0.963)	0.049	0.952 (0.697, 1.300)	0.758	–0.225 (–0.684, 0.234)	0.336	1.059 (0.731, 1.535)	0.762
Unexposed	*Ref.*				*Ref.*			
**Fgroup**								
Adolescent	0.490 (–0.559, 1.538)	0.360	0.680 (0.311, 1.488)	0.335	0.171 (–0.537, 0.879)	0.636	0.764 (0.418, 1.396)	0.381
School-age	0.144 (–0.551, 0.839)	0.685	0.874 (0.520, 1.468)	0.610	–0.036 (–0.559, 0.486)	0.891	1.068 (0.697, 1.638)	0.763
Infant	0.024 (–0.520, 0.568)	0.931	0.924 (0.617, 1.383)	0.700	0.179 (–0.310, 0.669)	0.473	0.921 (0.615, 1.378)	0.688
Fetal	–0.051 (–0.496, 0.393)	0.822	1.064 (0.766, 1.478)	0.712	–0.477 (–0.907, -0.047)	0.030	1.472 (1.044, 2.075)	0.027
Unexposed	*Ref.*				*Ref.*			
**Survey year: 2018**								
**Mgroup**								
Adolescent					0.020 (–1.334, 1.374)	0.977	0.594 (0.217, 1.627)	0.311
School-age					–0.165 (–1.015, 0.686)	0.704	0.719 (0.391, 1.321)	0.288
Infant					0.059 (–0.797, 0.914)	0.893	0.788 (0.422, 1.470)	0.454
Fetal					0.562 (–0.170, 1.293)	0.132	1.388 (0.832, 2.314)	0.209
Unexposed					*Ref.*			
**Fgroup**								
Adolescent					–0.049 (–1.170, 1.072)	0.931	0.935 (0.411, 2.126)	0.872
School-age					0.117 (–0.725, 0.959)	0.785	1.238 (0.685, 2.237)	0.480
Infant					0.305 (–0.501, 1.110)	0.458	1.050 (0.591, 1.865)	0.868
Fetal					–0.263 (–0.966, 0.439)	0.463	0.786 (0.472, 1.309)	0.356
Unexposed					*Ref.*			

*^‡^The data of the 2018 offspring was corrected using the basic information and their parents’ 2010 depression scores.*

*Adjusted odds ratios (aORs) and 95% confidence intervals (CIs) were calculated with multivariable logistic regression models to measure the association between parental famine exposure and outcomes (their late-life and offspring depression), while adjusting for current location, sex, education level, marital status, age in 2010, income level, social relation, happiness, social status, life satisfaction, body mass index (BMI), health status, diagnosed disease, smoking status, drinking status, and nap status. Mgroup and Fgroup refer to the famine group according to mother’s or father’s birth time, respectively. Mothers or fathers who were born 1946.1.1–1948.12.31 are classified as adolescent famine exposure; born 1949.1.1–1956.12.31 are classified as school-age famine exposure; born 1957.1.1–1958.12.31 are classified as infancy famine exposure; born 1959.1.1–1961.12.31 are classified as fetal famine exposure; born 1962.1.1–1969.12.31 are classified as control group.

*Control variables in multivariable linear regression analysis are the same as that in multivariable logistic regression models.*

*The score < 0 is depression and > 0 is normal in the CFPS, 2010 survey; the total score > 15 is depression, and ≤ 15 is normal in the CFPS, 2018 survey.*

Table 4 presents that compared with the unexposed group in 2010, parental fetal famine exposure was significantly associated with reduced their word test scores (mother: β = –1.614, *95% CI*: –2.535, –0.693; *p* = 0.001; father: β = –2.153, *95% CI:* –3.104, –1.202, *p* < 0.001) and the father’s infant famine exposure group was negatively related with their late-life word test scores (*p* = 0.024). The results also showed that compared with the unexposed group, maternal infant famine exposure was significantly associated with decreased mathematical calculation ability of offspring (*p* = 0.013). Compared with the unexposed group, maternal exposure to adolescent famine was significantly associated with lower word test score of offspring (*p* = 0.008). At the same time, the results in the table show that there were no statistically significant differences in the mathematical computing ability between the parental famine exposure group and the unexposed group, nor was there a difference in the literacy ability between the offspring paternal exposure groups and unexposed groups. The interaction of parental famine exposure had no statistically significant influence on the literacy ability of children.

**TABLE 4:** Association between famine-exposed parents and their offspring and their cognition scores in 2010.

	Math test		Word test	
	β (95%CI)	*P*	β (95%CI)	*P*
**Parental**				
**Mgroup**				
Adolescent	–0.101 (–1.135, 0.934)	0.849	1.949 (–0.189, 4.087)	0.074
School-age	–0.287 (–0.958, 0.385)	0.403	0.557 (–0.831, 1.945)	0.432
Infant	0.328 (–0.227, 0.884)	0.247	–0.995 (–2.143, 0.153)	0.089
Fetal	–0.151 (–0.597, 0.295)	0.507	–1.614 (–2.535, –0.693)	0.001
Control	*Ref.*		*Ref.*	
**Fgroup**				
Adolescent	–0.843 (–1.942, 0.255)	0.132	0.008 (–2.240, 2.256)	0.994
School-age	–0.695 (–1.423, 0.033)	0.061	–0.321 (–1.810, 1.169)	0.673
Infant	0.092 (–0.478, 0.663)	0.751	–1.349 (–2.516, –0.182)	0.024
Fetal	–0.381 (–0.846, 0.084)	0.108	–2.153 (–3.104, –1.202)	< 0.001
Control	*Ref.*		*Ref.*	
**Offspring^‡^**				
**Mgroup**				
Adolescent	–0.802 (–1.740, 0.135)	0.094	–2.346 (–4.067, –0.625)	0.008
School-age	–0.542 (–1.119, 0.035)	0.066	–0.322 (–1.381, 0.737)	0.551
Infant	–0.730 (–1.307, -0.153)	0.013	–0.141 (–1.199, 0.918)	0.794
Fetal	–0.300 (–0.802, 0.202)	0.242	–0.250 (–1.171, 0.671)	0.594
Control	*Ref.*		*Ref.*	
**Fgroup**				
Adolescent	–0.045 (–0.821, 0.731)	0.910	–0.440 (–1.864, 0.984)	0.545
School-age	0.261 (–0.309, 0.830)	0.369	–0.280 (–1.326, 0.766)	0.600
Infant	0.213 (–0.324, 0.749)	0.437	0.089 (–0.893, 1.072)	0.858
Fetal	–0.111 (–0.582, 0.360)	0.645	–0.662 (–1.526, 0.202)	0.133
Control	*Ref.*		*Ref.*	

*^‡^The data of offspring was corrected using the basic information and their parents’ cognitive scores.*

*Mgroup and Fgroup refer to the famine group according to mother’s or father’s birth time, respectively. Mothers or fathers who were born 1946.1.1–1948.12.31 are classified as adolescent famine exposure; born 1949.1.1–1956.12.31 are classified as school-age famine exposure; born 1957.1.1–1958.12.31 are classified as infancy famine exposure; born 1959.1.1–1961.12.31 are classified as fetal famine exposure; born 1962.1.1–1969.12.31 are classified as the control group.*

*Multiple linear regression model was adjusted by current location, sex, education level, marital status, age in 2010, income level, social relation, happiness, social status, life satisfaction, body mass index (BMI), health status, diagnosed disease, smoking status, drinking status, and nap status.*

**FIGURE 2:**
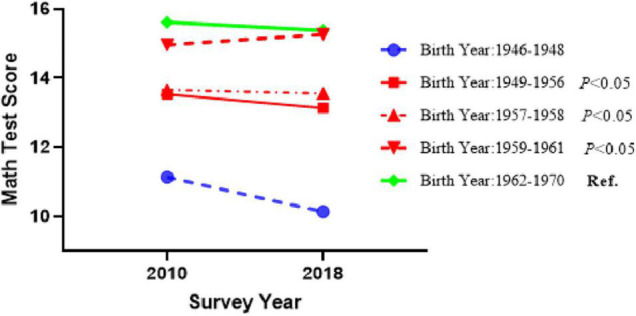
Differences in math test scores between the baseline measurement in 2010 and the follow-up measurement in 2018 between the famine exposure group and the unexposed group, adjusted for all the covariables listed in Table 1.

The differences of offspring cognitive function between the baseline measurement in 2010 and the follow-up measurement in 2018 between mother’s famine exposure group and the unexposed group was further analyzed. Figure 2 shows the differences in mathematical calculation ability score between the different famine exposure groups from 2010 to 2018. According to the mathematical calculation ability corresponding to maternal school-age famine exposure, infant famine exposure, and fetal famine exposure, the differences from 2010 to 2018 were significantly different compared with unexposed group (*p* < 0.05). Only the mathematical calculation ability of the offspring of the maternal fetal famine exposure group showed an upward trend, and the changes in the other groups showed a downward trend. However, no significant differences were found for word test (*p >* 0.05).

## Discussion

### Association of Parental Famine Exposure With Their Late-Life Cognitive Function

Our findings suggest that exposure to famine during the father’s fetal and infant period and mother’s fetal period was associated with their decreased word test scores compared with the unexposed population.

It is more likely for parents to be exposed to famine in early life, leading to reduce cognitive level, which is supported by many studies. Wang et al. found that fetal famine exposure was related to a low Mini-Mental State Examination score. They believed that fetal and childhood famine exposure were related to the decline of cognitive ability, which was reflected in selective attention and inhibition of response (27). Likewise, Wouters et al. reported that under different standards of exposure to famine, it was most likely that people who experienced famine during the fetal period, especially in the early stages of pregnancy, will experience cognitive decline (28). Rong et al. observed that exposure to fetal famine was associated with a high risk of overall cognitive decline (29, 30), and De Rooij et al. found that the male brain volume in the fetal famine exposure group was smaller, and malnutrition during this period would have a permanent impact on brain volume (31). Moreover, our results showed that not only the fetal stage, but also infant stage might the crucial period for brain development. However, some studies have shown that prenatal famine exposure does not seem to be associated with cognitive dysfunction. Arage et al. reported that postpartum, rather than prenatal famine exposure was significantly negatively correlated with adult cognitive function (32).

The current speculation about the association between parental famine exposure in the fetal and infant period and the risk of cognitive impairment focuses on the impact of famine during a key window period of organ development (28, 31). The structure of the central nervous system in the brain is formed during early pregnancy. When famine occurs in this period, malnutrition may hinder the development of the central nervous system, and affect the size of the head circumference and the brain capacity of men, thereby hindering the development of cognitive function in later periods (33). Adult rats born to dams fed a low-protein diet during pregnancy and lactation exhibited impaired encoding and consolidation of memory. It was related to reduced production of newly born hippocampal neurons (34).

### Association of Parental Famine Exposure With Offspring Depression and Cognitive Function

Our study aimed to explore the intergenerational association of famine exposure with depression and cognitive levels. It is not difficult to see from the summary results that compared with the unexposed group, the father’s fetal famine exposure was associated with increased depression risk in their offspring in 2010, however, a significant correlation was not found in 2018.

At present, most research has focused on the role of the mother on offspring health, there are few studies concerning father fasting. A growing body of evidence suggests that paternal nutrition is predictors for the development of psychopathology in offspring (35, 36). One possibility is that deleterious effects issued from fathers are transmitted to the next generations via small non-coding RNAs (sncRNAs) present in the sperm (36). Van Steenwyk used a paternal post-natal trauma mouse model and reported that depression-like behaviors were inherited intergenerationally through the fathers, and used the model to prove that sperm RNA was genetically dependent on lineages (37), which might explain why we found that paternal exposure to fetal famine affected offspring’s depression levels. In addition, malnutrition is the most common disease in famine areas, such as vitamin and mineral deficiencies (38), where the role of vitamin D is widely considered (39). In the same context, Tolppanen et al. found that insufficient level of vitamin D in childhood was associated with the risk of depression in later life. In the long term, vitamin D supplementation may have a protective effect on depression by alleviating the effects of depression caused by exposure to famine (40). This may explain why, in our findings, the significant correlation between father’s fetal famine exposure and cognitive function disappeared in 2018.

Our research results show that maternal infant and adolescent famine exposure was significantly associated with decreased cognitive ability of the offspring. Li et al. held the same view that there was no significant correlation between parental prenatal hunger exposure and the cognitive function of adult offspring, after controlling for confounding factors (41). The participants of their research were selected from Heilongjiang province located northeast of China, which suffered from the same severe famine from 1959 to 1961. With regard to the intergenerational transmission of famine, more studies have focused on parental prenatal exposure to famine, but not in the field of postnatal exposure. A possible mechanism involves epigenetic modifications on the sperm or egg (42). Previous studies have presents that limited food availability during pre-adolescence leads to worse pubertal development and epigenetic modifications on the sperm or egg (42). While the modifications are inherited through the products of conception and are directly inherited by future generations (43).

### Limitations

Taken together, our research focused on the association of parental famine exposure with offspring cognitive function and depression, which is an area rarely covered by current research. Moreover, the data are based on a large representative sample survey, covering most provinces and cities in China, using face-to-face communication data collection and data with high reliability. The limitations of this study are as follows: (1) there is no data on the severity of the famine, and it is impossible to establish a relationship between the degree of famine experienced by the respondents and depression and cognitive function; (2) not all characteristics that might influence depressive symptoms and cognitive function, such as stress or a dramatic change in lifestyle, were measured, which might confound the interpretation of the relationship between famine exposure and outcomes; (3) the CFPS data did not include the explanatory factors on epigenetic factors or vitamin D, and therefore it is difficult to establish the mechanism linking famine and depression or cognitive function through epigenetic or vitamin D; (4) this paper was unable to explore the causal relationship between exposures and outcomes. Further studies are needed to account for these limitations.

## Conclusion

This study examined the relationship between parental famine exposure and depression and cognition in the offspring. One of the more significant findings to emerge from this study is that parents experience famine in utero or early in life was associated with decreased cognitive function. We find that father’s fetal famine exposure was associated with increased depression risk in their offspring. Maternal infant and adolescent famine exposure was significantly associated with decreased cognitive ability of the offspring. More research is needed to explain the intergenerational transmission mechanisms of famine.

## Data Availability Statement

The original contributions presented in the study are included in the article/supplementary material, further inquiries can be directed to the corresponding authors.

## Ethics Statement

The studies involving human participants were reviewed and approved by the Peking University Biomedical Ethics Review Committee provided ethical approval for the survey (Approval number: IRB00001052-14010). Written informed consent to participate in this study was provided by the participants’ legal guardian/next of kin.

## Author Contributions

YeL contributed to the data analysis, writing original draft, and modifying manuscript. YuL and YZL contributed to the literature search and modifying manuscript. SH and JL contributed to conceptualization, data analysis, modifying manuscript and supervision.

## Conflict of Interest

The authors declare that the research was conducted in the absence of any commercial or financial relationships that could be construed as a potential conflict of interest.

## Publisher’s Note

All claims expressed in this article are solely those of the authors and do not necessarily represent those of their affiliated organizations, or those of the publisher, the editors and the reviewers. Any product that may be evaluated in this article, or claim that may be made by its manufacturer, is not guaranteed or endorsed by the publisher.
